# The relation between surface acidity and MoO**_3_**:Al**_2_**O**_3_** ratio on the ternary mixed oxide catalysts for the conversion of propan-2-ol

**DOI:** 10.55730/1300-0527.3505

**Published:** 2022-10-08

**Authors:** Hani ZEIDAN, Ebru ERÜNAL, Mustafa Esen MARTI

**Affiliations:** 1 Department of Chemical Engineering, Konya Technical University, Konya, Turkey; 2 Department of Chemical Engineering, Çukurova University, Adana, Turkey

**Keywords:** Catalyst, mixed oxides, propene, surface acidity, ternary system

## Abstract

In this study, ternary mixed oxide catalysts containing Al_2_O_3_-MoO_3_-MgO and Al_2_O_3_-MoO_3_-WO_3_ were prepared with a changing ratio of MoO_3_:Al_2_O_3_ between 0.05 and 20.00. All catalysts showed 100% selectivity towards propene during the conversion of propan-2-ol at temperatures between 220 and 400 °C. The catalysts prepared from WO_3_ possessed very strong acid sites, which cause higher catalytic activity than catalysts prepared from MgO. Besides, the ratio of MoO_3_:Al_2_O_3_ was found to be directly proportional to the conversion yield for all catalysts. XRD results show that whole MgO reacted with Al_2_O_3_ and MoO_3_ to form amorphous MgMoO_4_ and MgAl_2_O_4_ phases during catalyst preparation. Furthermore, WO_3_ reacted only with Al_2_O_3_ to form Al_2_(WO_4_)_3_ and WO_3_ phase was also detected in the final product. The higher surface acidity and catalytic activity of Al_2_O_3_-MoO_3_-WO_3_ catalyst referred to this WO_3_ phase within the structure.

## 1. Introduction

One of the most widely manufactured polymers in industry is polypropylene (PP). Its global market size was around USD 117.8 billion in 2020 and is projected to increase at an annual growth rate of 3.4% from 2021 to 2028 [1]. PP is synthesized via polymerization reaction of propene molecules and high purity of the monomers used in the process is strictly required for an efficient process. Practically 2/3 of the propene in industry is used for the production of PP [2]. Besides, it is employed for the manufacture of propene oxide (epoxypropane), acrylic (propenoic) acid, acrylonitrile (propenenitrile), butanol, and (1-Methylethyl) benzene (cumene) [1–3].

Propene is currently produced as a by-product fluid catalytic cracking and coproduct of naphtha catalytic cracking [3]. Propene obtained from refineries can be used in liquefied petroleum gas or to enhance the octane number in gasoline [1,2]. However, even negligible amounts of impurities can prevent the polymerization of propene into polypropylene. In addition, current production methods use huge amounts of energy, which also negatively influenced the greenhouse emissions [4,5].

Propene can be alternatively produced by catalytic dehydration of propan-2-ol (Eq. 1) [6]. In addition, this reaction is a probe to determine the acid-base sites of the solid catalysts [7]. In this context, the presence of acid sites (Brønsted and/or Lewis) on the catalyst lead propene formation by dehydration, whereas acetone is formed via the dehydrogenation of propanol-2-ol (Eq.2) in the presence of basic sites or acid-base couples [3,8–17].


(1) $$CH_{3}-CHOH-CH_{3}\to CH_{3}-CH=CH_{2}+H_{2}O$$

(2) $$CH_{3}-CHOH-CH_{3}\to CH_{3}-CO-CH_{3}+H_{2}$$

In the literature, mainly supported catalysts have been employed for the dehydrogenation of propan-2-ol while various metal oxide catalysts were studied for the catalytic dehydration of propan-2-ol [9–22]. For example, Cu/Al_2_O_3_ catalysts were used for the selective dehydrogenation of propan-2-ol to form acetone [10]. Moreover, high selectivity was obtained with Pt/ZrO_2_ catalyst at the temperatures lower than 250 °C (T < 250 °C) for acetone formation [11]. Au/CeO_2_ catalysts were reported to enhance the selectivity towards acetone by suppressing the dehydration of propan-2-ol [12]. Lately, CuO_x_PtO_x_/TiO_2_-ZrO_2_ catalysts showed high efficiency and selectivity due to their basic properties [13]. Supports are known to increase mechanical stability of a catalyst by dispersing the catalytically active sites on the catalyst surface. However, in mass production, important reactions are still carried out with unsupported catalysts such as iron catalysts used in Haber process. Therefore, for the dehydrogenation of propan-2-ol, developing unsupported catalysts are inevitable to be used in industrial production.

Studies on the use of unsupported V_2_O_5_ catalyst and its modified versions for the decomposition of propan-2-ol showed a close relationship between the surface acidity and catalytic activity [9]. Similarly, Zr(HPO_4_)_2_.nH_2_O catalysts on the conversion of propan-2-ol were also recorded to be effective with total conversion and selectivity toward propene of 100% [14]. In addition, mixed forms of Al_2_O_3_ and TiO_2_ were shown to be more effective than their single forms and the amount of surface acidity was observed to have a stronger effect than the strength of surface acidic sites [15]. Moreover, sulfate content was reported to increase the acidic sites on the surface of the catalysts and therefore, higher propene selectivity was achieved [16].

Al_2_O_3_ and MoO_3_ are known to possess acidic active sites for various types of acid-catalyzed reactions [18–20]. However, the impact of their ratio in a ternary mixed oxide catalyst was not systemically studied yet. The addition of WO_3_ to Al_2_O_3_/MoO_3_ enhances the acidic properties of the surface [21]. On the other hand, MgO addition may result in the appearance of basic sites on the surface which would enhance acetone formation [22]. Therefore, in the present study, the catalytic activity and the effects of MoO_3_:Al_2_O_3_ ratio, surface acidity and the type of acid sites on the selective dehydration of propan-2-ol to form propene were investigated for a series of Al_2_O_3_-MoO_3_-MgO (AMMO) and Al_2_O_3_-MoO_3_-WO_3_ (AMWO) catalysts at various temperatures.

## 2. Experimental

### 2.1. Materials

The following chemicals were used for the synthesis of the catalysts: Aluminum nitrate nonahydrate (Al(NO_3_)_3_.9H_2_O; ≥ 98.5%; CAS 7784-27-2), ammonia solution (NH_3(aq)_; 25.0%; CAS 7664-41-7), calcium chloride (CaCl_2_; ≥98.0%; CAS 10043-52-4), nitric acid (HNO_3_; 65.0%; CAS 7697-37-2), magnesium nitrate hexahydrate (Mg(NO_3_)_2_.6H_2_O; 99.99%; 13446-18-9), n-butylamine (CH_3_(CH_2_)_3_NH_2_; ≥ 99.0%; CAS 109-73-9), ammonium heptamolybdate ((NH_4_)_6_Mo_7_O_24_; 99.3-101.8%; CAS 12054-85-2), and ammonium paratungstate ((NH_4_)_10_(H_2_W_12_O_42_).4H_2_O; 99.99%; CAS 11120-25-5) were purchased from Merck Co. Acetonitrile (CH_3_CN; 99.0%; CAS 75-05-8), and pyridine (C_5_H_5_N; 99.5%; CAS 110-86-1) were supplied from Scharlau Co. All chemicals were used without any treatment.

### 2.2. Synthesis of the catalysts

The catalysts were synthesized according to the methods given in references [23–26]. The target catalyst compositions and symbols are summarized in Table 1.

Firstly, the precursors of the metal oxides were prepared as follows: (NH_4_)_6_Mo_7_O_24_ (ammonium heptamolybdate), Mg(NO_3_)_2_.6H_2_O (magnesium nitrate hexahydrate), (NH_4_)_10_(H_2_W_12_O_42_).4H_2_O (ammonium paratungstate), (NH_4_)_10_(H_2_W_12_O_42_).4H_2_O (ammonium paratungstate) and Al(NO_3_)_3_.9H_2_O (aluminium nitrate nonahydrate) were dissolved in the least amount of bidistilled water, separately. The pH of the solutions was adjusted at 6 with the addition of 25% ammonia- or 1% nitric acid solutions under continuous stirring for 4 h. All precursors were prepared at room temperature except the tungsten precursor, which was prepared at 323 K. All precursors were in liquid phase except the alumina precursor, which was in gel phase after kept at room temperature overnight. These precursors were then mixed in appropriate amounts (Table 2) to obtain the reactions given in Eqs. 3 and 4. Firstly, Al(OH)_3_ and (NH_4_)_6_Mo_7_O_24_ were mixed and then either (NH_4_)_10_(H_2_W_12_O_42_)·4H_2_O or Mg(NO_3_)_2_·6H_2_O were added to the solution. All reactions were conducted at room temperature under continuous stirring for 4 h.


(3) $$Al{\left(OH\right)}_{3}+{\left(NH_{4}\right)}_{6}Mo_{7}O_{24}+Mg{\left(NO_{3}\right)}_{2}.6H_{2}O\to 1/2{Al}_{2}O_{3}+7MoO_{3}+MgO+2NO_{2}+6NH_{3}+21/2H_{2}O+1/2O_{2}$$

(4) $$Al{\left(OH\right)}_{3}+{\left(NH_{4}\right)}_{6}Mo_{7}O_{24}+{\left(NO_{4}\right)}_{10}(H_{2}W_{12}O_{42}).4H_{2}O\to 1/2{Al}_{2}O_{3}+7MoO_{3}+12WO_{3}+16NH_{3}+29/2H_{2}O$$

The obtained AMMO and AMWO catalysts were dried at 120 °C overnight. They were then calcined first at 300 °C for 5 h and at 500 °C for another 4 h. The temperature was gradually raised (10 °C min^−1^) from 300 °C to 500 °C. Finally, the samples were placed in a glass dryer containing anhydrous calcium chloride and cooled to room temperature before crushed in a mortar.

### 2.3. Characterization

The XRD patterns of the calcined catalysts (at 500 °C) were determined using a Philips Diffractometer Type PW 1830. The patterns were run with nickel-filtered Cu-K α radiation (λ = 1.5418 Å) at a voltage and current of 30 kV and 30 mA, respectively. The analyses were carried out in the range of 20° ≤ 2θ ≤ 80°. The FT-IR spectra of the catalysts were obtained with a Bruker-Vector 22 spectrophotometer in the range of 525 and 4000 cm^−1^. The samples were prepared by mixing 0.005 g sample with 0.1 g KBr in 10 mm diameter self-supporting disks. The microstructures of the catalysts were investigated via a JSM-7610F Schottky Field Emission Scanning Electron Microscope (SEM). The elemental compositions of the phases were analyzed by a Jeol JSM 5410 LV coupled with an Oxford Energy Dispersion Spectrometer (EDS) equipped with a Link-Isis software.

### 2.4. Measurement of surface acidity

Firstly, 0.2 g dry sample was suspended in 20 mL acetonitrile and stirred for 4 h during the titration. The suspension was then titrated with a solution of n-butylamine (0.005 mol L^−1^) in acetonitrile at a flow rate of 0.1 mL min^−1^. The electrode potential change was determined using a digital double (pH–mV) junction electrode (Inolab, WTW). Addition of the amine continued until the electrode potential (mV) stabilized. The Brønsted and Lewis acidic sites on the surface of the catalysts were identified by adsorption-desorption of pyridine [27]. The catalysts were initially degassed at 200 °C for 3 h under high vacuum followed by saturating with pyridine. Next, the excess pyridine was evaporated at 70 °C overnight. Finally, the dried samples were subjected to FT-IR spectra analysis.

The initial electrode potential value (Ei, mV) is recommended to depict the most strong acidic sites. The potential value denotes the number of total acidic sites (mmol amine/g solid) at the plateau of the titration curve. The strength of the acidic sites can be classified according to the E_i_ values as: 1) very strong sites (E_i_ > 100 mV), 2) strong sites (0 < E_i_ < 100 mV), 3) weak sites (−100 < E_i_ <0), 4) very weak sites (E_i_ < −100 mV) [28,29].

### 2.5. Catalytic activity

The catalytic activity of the catalysts was tested for the conversion of propan-2-ol into propene using a microcatalytic pulse technique. The microcatalytic reactor was coupled with a gas chromatography (Shimadzu, GC-9A), which was also equipped with a flame ionization detector. The temperature of the reactor was monitored by using a K-type thermocouple contacted with the reactor wall. The catalysts were activated at 400 °C for 3 h with a stream of pure nitrogen gas and a flow rate of 30 mL min^−1^. Next, 0.02 g of each catalyst was placed in the reactor tube and supported by quartz wool. Afterwards, 0.1 μL of propan-2-ol was injected on the activated catalysts and the products were analyzed by GC. The reaction was investigated in the temperature range of 220–400 °C.

## 3. Results and discussion

### 3.1. Catalyst characterization

**Figure 1:**
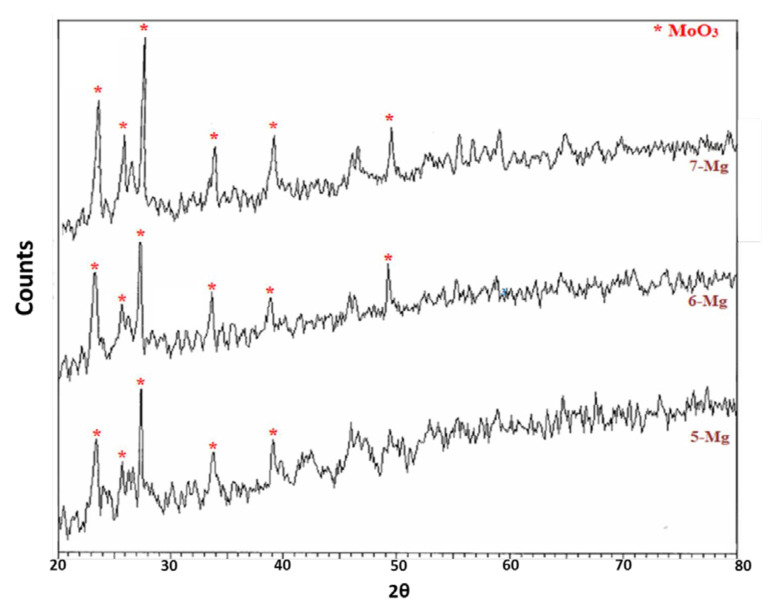
X-ray diffraction patterns of the MgO including (AMMO) catalysts.

XRD patterns of the AMMO samples are given in Figure 1. In general, Al_2_(MoO_4_)_3_, MgMoO_4_, and MgAl_2_O_4_ (spinel) phases are expected to form due to the calcination of MgO at 500 °C. Hence, the first reaction occurs between Al_2_O_3_ and MoO_3_ (Eq. 5) while the second between MgO and MoO_3_ (Eq. 6). Finally, the third reaction takes place between Al_2_O_3_ and MgO (Eq. 7). Since no extra peak for MgO was detected, all MgO seems to be consumed during the calcination.


(5) $$3MoO_{3}+{Al}_{2}O_{3}\to {Al}_{2}{\left(MoO_{4}\right)}_{3}$$

(6) $$MgO+MoO_{3}\to MgMoO_{4}$$

(7) $${Al}_{2}O_{3}+MgO\to Mg{Al}_{2}O_{4}$$

The expected peaks for Al_2_(MoO_4_)_3_, MgMoO_4_, and MgAl_2_O_4_ (spinel) phases cannot be detected in Figure 1 [30–35]. However, only the characteristic peaks of MoO_3_ phase could be identified for the 2.00-Mg, 4.00-Mg, and 20.00-Mg samples [31]. Especially when Mg ratio is higher, sharper MoO_3_ peaks at 23.4°, 25.7°, 27.5°, 34°, 39°, and 49° show that the reaction according to Eq. 7 rather than Eq. 6 so that excess MoO_3_ peaks were detected. Moreover, peaks at 23.4° and 25.7° were reported for Al_2_(MoO_4_)_3_ phase [36]. On the other hand, the absence of MgAl_2_O_4_ peaks indicates that the obtained products must be in amorphous phase.

**Figure 2:**
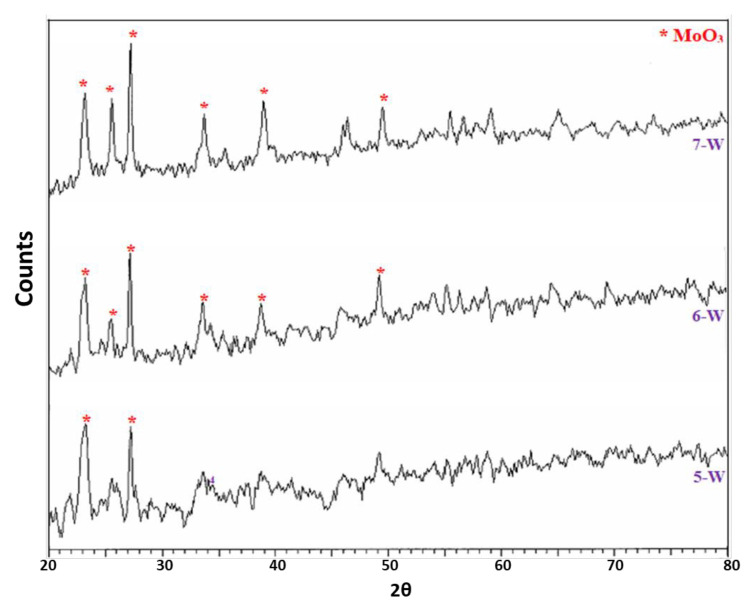
X-ray diffraction patterns of the WO_3_ including (AMWO) catalysts.

XRD patterns of AMWO samples are given in Figure 2. Similarly, the dominating phase seems to be MoO_3_ for AMWO samples. Especially for the 2.00-W, 4.00-W, and 20.00-W samples, the characteristic peaks of MoO_3_ and Al_2_(MoO_4_)_3_ phases are clearly detected [31]. This also shows that most of W-including phases are in amorphous phase. When W amount decreases, peak at 49° corresponding to MoO_3_ phase disappears. It is probably due to the reaction between MoO_3_ and Al_2_O_3_ shown in Eq. 5.


(8) $${Al}_{2}O_{3}+3WO_{3}\to {Al}_{2}{\left(WO_{4}\right)}_{3}$$

The XRD analysis showed that when MoO_3_:Al_2_O_3_ ratio is equal to or higher than 2.00 (2.00-Mg, 4.00-Mg, 20.00-Mg and 2.00-W, 4.00-W, 20.00-W), the expected phases formed. Otherwise, the crystallinity is not observed (0.25-Mg, 0.50-Mg, 1.00-Mg & 0.25-W, 0.50-W, 1.00-W) due to the materials stay in amorphous phase (0.05-Mg and 0.05-W). The calcination temperature at 500 °C may not be sufficient for the formation of crystal phases. Hence, the formation of crystalline phases upon 600 °C for similar compounds was previously mentioned in the literature [36].

**Figure 3:**
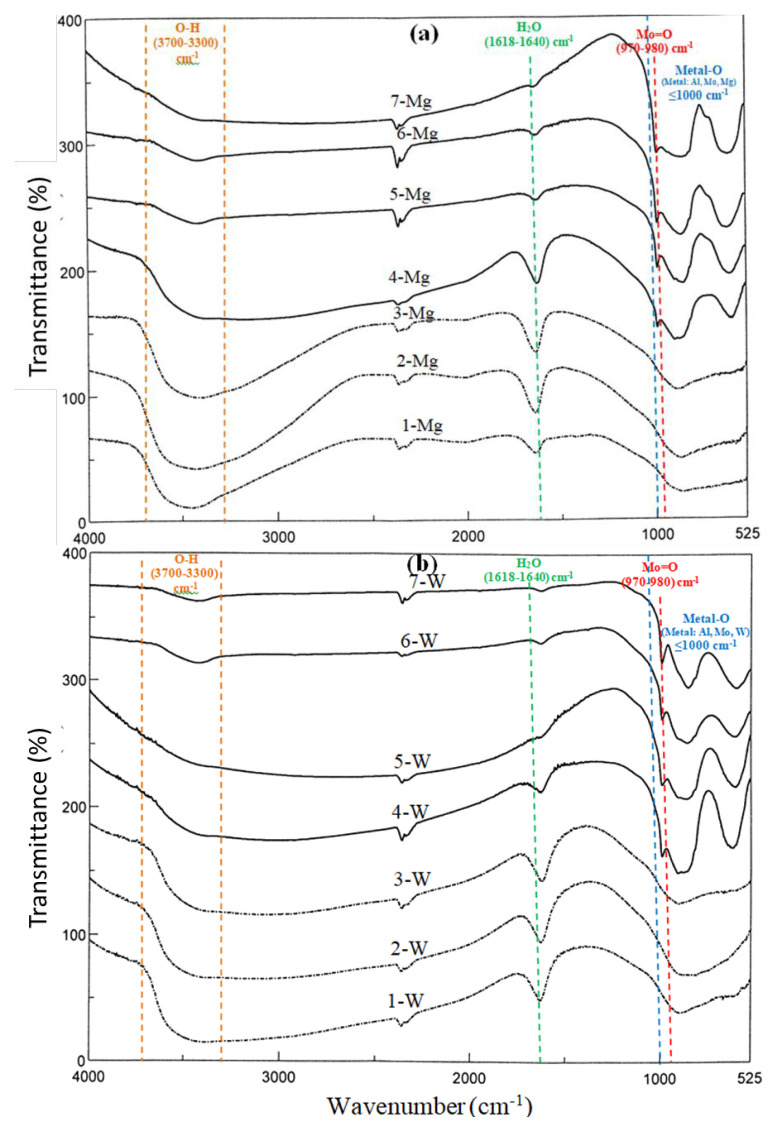
FT-IR spectra of the a) MgO including (AMMO) and b) WO_3_ including (AMWO) catalysts.

The FT-IR spectra of the prepared samples have similar features except the intensities of the peaks (Figure 3). The band at 3700–3300 cm^−1^ was associated to the hydroxyl groups while the peak appeared at 1618–1640 cm^−1^ was attributed to adsorbed water. The FT-IR spectra of the 1.00-W, 2.00-W, 4.00-W, 20.00-W, 1.00-Mg, 2.00-Mg, 4.00-Mg, and 20.00-Mg samples indicated the stretching vibration of Mo=O bonds at 970–980 cm^−1^ [37]. The vibration of Mo-O bonds and (Mo-O-Mo) in MoO_3_ were observed at 850–900 cm^−1^ and 590–630 cm^−1^, respectively.

The broad peak observed with the 1.00-W, 2.00-W, 4.00-W, and 20.00-W samples around 686 cm^−1^ were assigned to the (W–O–W) bridging vibration of the corner-sharing WO_6_ octahedron in the WO_3_ crystal. On the other hand, the band about 548 cm^−1^ could be attributed to the Mg=O bending vibration in the 1.00-Mg, 2.00-Mg, 4.00-Mg, and 20.00-Mg samples. These results, observations, and trends are consistent with the reports in the literature [38,39].

In addition to these peaks, several interferences were observed in the range of ≤1000 cm^−1^. These were due to the appearance of metal-oxygen peaks in this field, which is also consistent with the literature [40].

**Figure 4:**
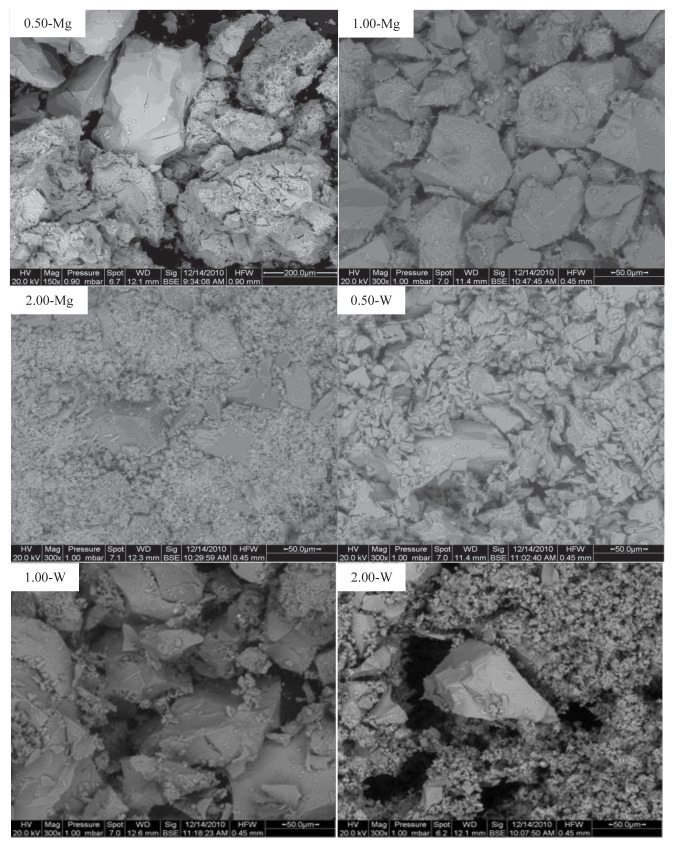
SEM images of the synthesized catalysts.

The SEM images of the catalysts, in Figure 4, show the formation of irregular aggregates whose dimensions are ranging between 50 and 200 μm. The aggregation formation was attributed to the applied heat during the calcination process [41]. Even though MoO_3_ phase was detected for 2.0-Mg and 2.0-W samples with XRD measurements, no significant change in the surface morphology with the different MoO_3_:Al_2_O_3_ ratios can be tracked through SEM images.

**Figure 5:**
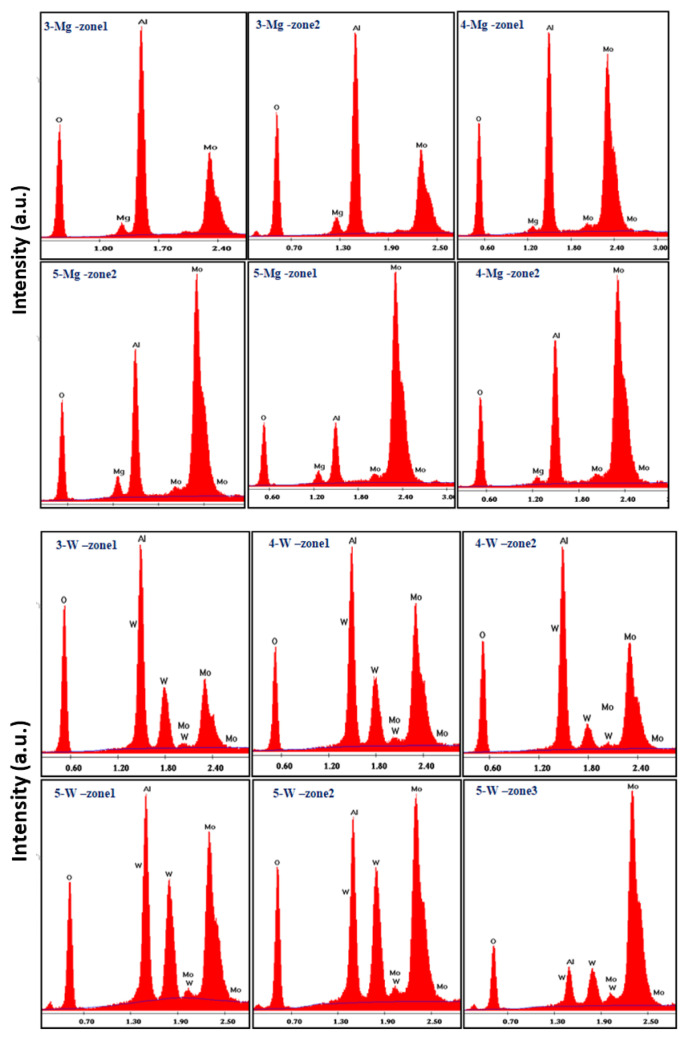
Intensity-energy loss plots of MgO including (0.50-Mg, 1.00-Mg, 2.00-Mg) and WO_3_ including catalysts (0.5-W, 1.00-W, 2.00-W).

The intensity-energy (I–E) plots obtained from EDS analysis were used to confirm the presence of Al, Mo, O and Mg or W elements in the samples (Figure 5). In addition, percentages of these elements were calculated. Based on the EDS results, the catalysts are free of either nitrate ions or any other possible impurities. The weight percentages (wt.%) of the elements within the samples are summarized in Table 2. The targeted molar ratios of the elements according to Table 1 were verified in the basis of measured elements via EDS 1. Moreover, MgO seems to react completely while WO_3_ reacted partially and the rest remained without any interaction. This is most probably since MgO reacts with both MoO_3_ and Al_2_O_3_ while WO_3_ only reacts with Al_2_O_3_ and WO_3_

### 3.2. Surface acidity

The acidic properties of the catalyst surfaces were investigated to interpret the catalytic activity for the conversion of propan-2-ol to propene. In general, the strength of the acidic sites of the solid catalysts is determined via titration with amines and with the help of special indicators (Hammett indicators) in anhydrous media such as dry benzene, acetonitrile, and cyclohexane [42]. In this study, the surface acidities were measured by potentiometric titration with a solution of n-butylamine in acetonitrile [25]. Since n-butylamine is a strong base, it can adsorb on both Brønsted or Lewis types of acidic sites with different strengths [28,29,43,44].

**Figure 6:**
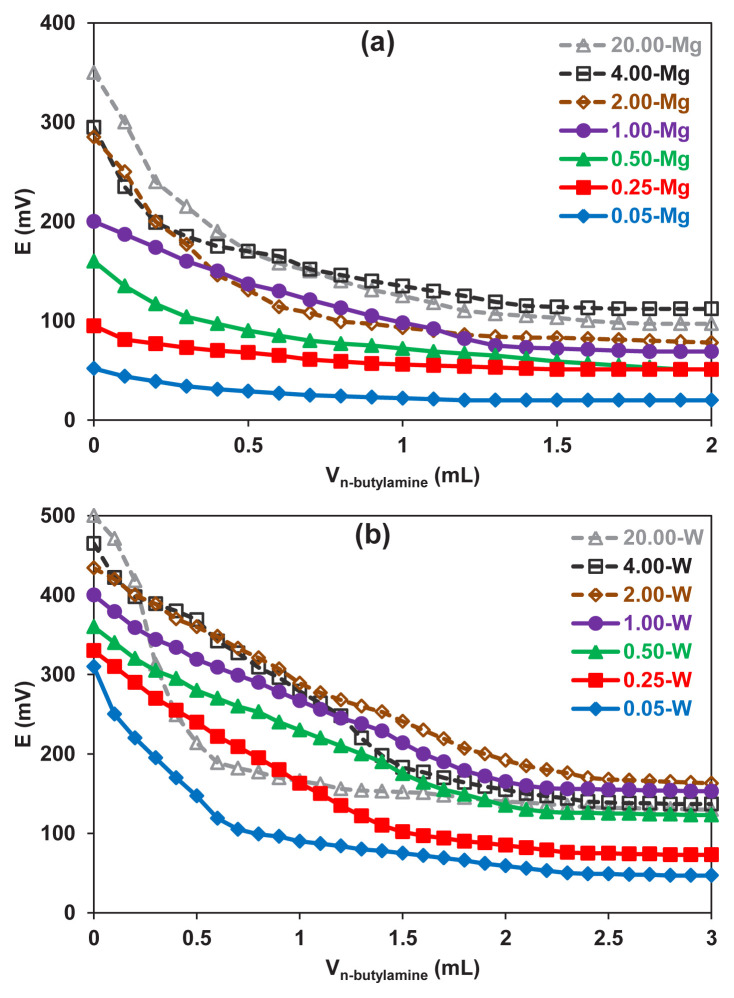
Potentiometric titration curves of catalysts a) AMMO and b) AMWO.

The E_i_ values in Figure 6 show that there are very strong acidic sites on the catalysts except for 0.05-Mg and 0.25-Mg. The acidic sites on these two samples are less strong acidic ones. It is also worth noting that the AMWO samples have stronger acidic sites (310 mV ≤ E_i_ ≤ 500 mV) than the AMMO catalysts (52 mV ≤ E_i_ ≤ 350 mV). WO_3_ phase might increase the surface acidity. A bulk phase analysis would help to track WO_3_ phase.

**Figure 7:**
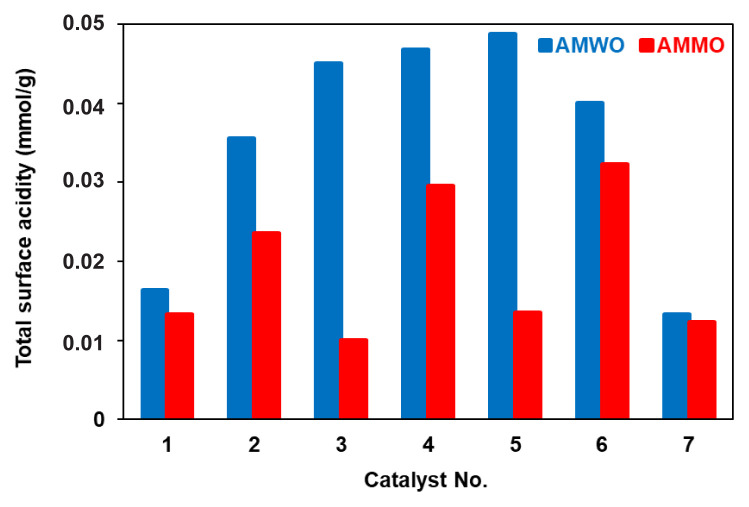
Variation in total surface acidity as a function of MoO_3_:Al_2_O_3_ ratio for MgO including (AMMO) and WO_3_ including (AMWO) catalysts.

Moreover, the strength of the acidic sites significantly increased when the molar ratio of MoO_3_:Al_2_O_3_ was increased from 0.05 to 20 at a fixed amount of WO_3_ or MgO for both samples. The total surface acidity of the solids strongly depends on the composition of the catalysts (Figure 7). An oscillation was observed for AMMO catalysts. The existence of MgO, which has acidic and basic properties, may cause this behavior [22]. For the AMWO system, the surface acidity enhanced with increasing MoO_3_:Al_2_O_3_ from 0.05 to 2 and reached 0.0488 mmol g^−1^ as the maximum value. However, increasing the molar ratio to 20 sharply reduced the surface acidity to 0.0133 mmol g^−1^. On the other hand, the maximum value (0.0323 mmol g^−1^) was obtained when the molar ratio of MoO_3_:Al_2_O_3_ was 4 for the AMMO system.

**Figure 8:**
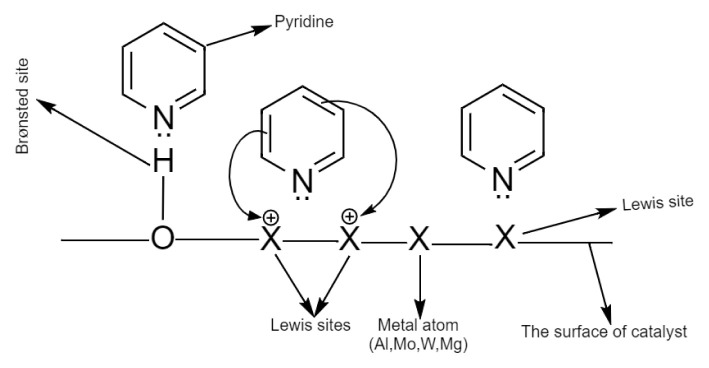
The chemisorption mechanisms of pyridine on Brønsted and Lewis acidic sites.

The type of the acidic sites (Brønsted and Lewis) on the surface was determined by the FT-IR spectra of the pyridine-adsorbed surface of the related catalyst [27]. Pyridine can be chemically adsorbed in various ways (Figure 8): 1) It is bounded by hydrogen bonding that refers to Brønsted acid sites and appears in the FT-IR spectra as the bands in the range of 1400–1447 cm^−1^, 2) It is bounded by coordinate covalent bonds due to the attraction of the electron pair of the nitrogen atom to the Lewis site on the catalyst surface and appears in the range of 1447–1460 cm^−1^, 1448–1503 cm^−1^, and 1600–1633 cm^−1^, 3) Due to its adsorption on the Brønsted sites, the pyridinium anion can appear in the range of 1485–1500 cm^−1^, 1540 cm^−1^, approximately 1620 cm^−1^ and approximately 1640 cm^−1^ [27].

**Figure 9:**
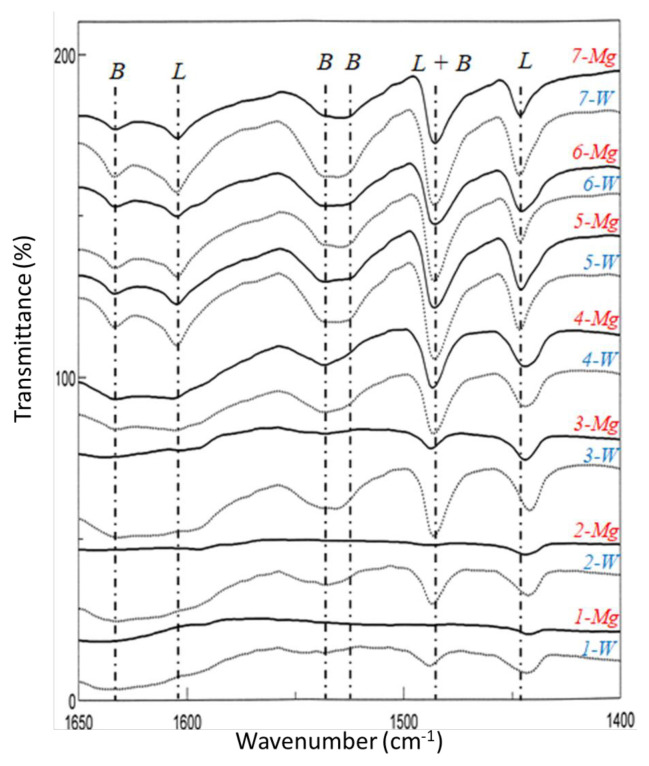
FT-IR spectra of pyridine adsorbed on the surface of the synthesized catalysts.

The FT-IR spectra of the pyridine-adsorbed catalysts are given in the range of 1400–1650 cm^−1^ (Figure 9). The intensities of all peaks belong to the pyridine-adsorbed surface increases with the molar ratio of MoO_3_:Al_2_O_3_. Type 1 adsorption as a result of hydrogen bonding around approximately 1447 cm^−1^ was detected for the samples whose MoO_3_:Al_2_O_3_ molar ratio was between 1 and 20 [43,45]. The bands shifted to the ≤1442 cm^−1^ when the molar ratio of MoO_3_:Al_2_O_3_ decreased below 1 for both systems. These shifts may indicate a weak adsorption on the acidic sites of either Brønsted or Lewis type [46]. A characteristic band appeared at 1486 cm^−1^ might most likely due to the adsorption of pyridine on Brønsted or/and Lewis acid sites [47,48]. The pyridine adsorbed on Brønsted acid sites that form pyridinium ion appeared as two bands at 1537 and 1633 cm^−1^ [28,42,43,47]. The band and shoulder at 1605 and 1525 cm^−1^ were attributed to the adsorption on the Lewis acid sites [28,29,42]. The bands detected for AMWO system were more intense than those noticed for AMMO system.

### 3.3. Catalytic activity

The conversion of propan-2-ol is known to take place with three parallel reactions [3,8–17]. The first one is the dehydration of propan-2-ol to obtain the propene (olefin) in the vicinity of catalysts with strong acid sites. The second one is the dehydrogenation of propan-2-ol to acetone because of the strong base sites of the catalysts or in the redox couple. The third one is the formation of isopropyl ether that preferentially depends on the number of acid sites than their strength.

**Figure 10:**
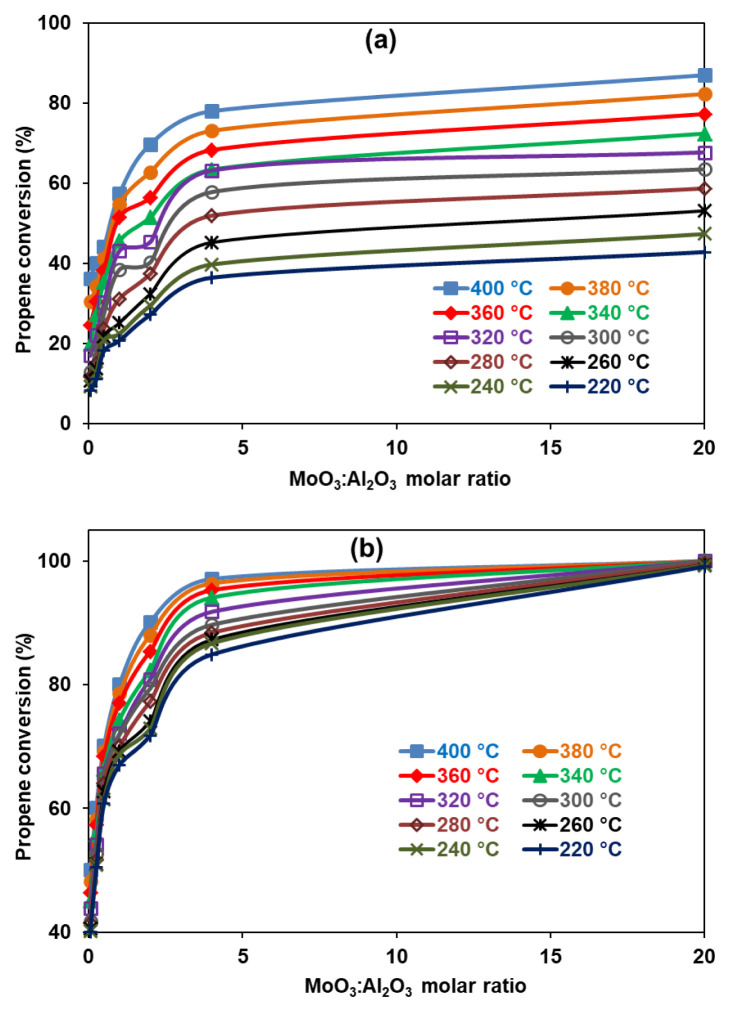
Effect of MoO_3_:Al_2_O_3_ ratio on the propene conversion of the dehydration of propan-2-ol reaction with a) AMMO and b) AMWO catalysts.

In the present study, the conversion of propan-2-ol leads only to the propene indicating a selectivity of 100% in the temperature range of 220–400 °C. This verifies the strong acid sites found via surface acidity investigations and the purity analyses obtained with EDS measurements. Even though it was reported that impurities in reactant decrease the selectivity towards propene, obviously, impurities that decrease the surface acidity would cause a similar effect [49]. Hence, the purity analyses of the catalysts confirmed it. In addition to these findings, it was exploited that the total conversion depends substantially on the molar ratio of MoO_3_:Al_2_O_3_. The conversion yield increased with the molar ratio of MoO_3_:Al_2_O_3_ for all catalysts, and the order was as follows: 0.05-Mg< 0.25-Mg< 0.50-Mg< 1.00-Mg< 2.00-Mg< 4.00-Mg< 20.00-Mg and 0.05-W< 0.25-W< 0.5-W< 1.00-W< 2.00-W< 4.00-W< 20.00-W for AMMO (Figure 10a) and AMWO (Figure 10b) systems, respectively. It can directly be related to the increasing ratio of MoO_3_ or decreasing Al_2_O_3_ amount. It verifies the potentiometric titration analysis in which strength of acidity was also found as a function of MoO_3_. Hence, a significant increase in acidic sites was observed when MoO_3_:Al_2_O_3_ ratio was increased. The highest yield (100%) and selectivity (100%) were obtained with the 20.00-W catalyst for all reaction temperatures. However, a high yield would also be expected when MoO_3_:Al_2_O_3_ ratio would be between 4 and 20. The conversion percentages on AMWO catalysts were much higher than the ones obtained on AMMO catalysts. This was most likely due to the presence of much stronger acidic sites on the former catalysts. The potentiometric titration results already showed that the probable presence of WO_3_ increased surface acidity. In addition to these, when MoO_3_:Al_2_O_3_ ratio was below 2, conversion reaches up to 50% (max.) at the highest temperature for AMMO catalysts while it reaches 70% for AMWO. Above this ratio, the conversions increase rapidly as a function of temperature. Especially for AMWO catalysts, nearly 70% conversion was recorded at 260 °C for the 2.0-W sample. The intense bands for AMWO catalysts obtained via FT-IR measurements already showed the presence of Lewis acid sites that would favor in the higher yields for this reaction.

**Figure 11:**
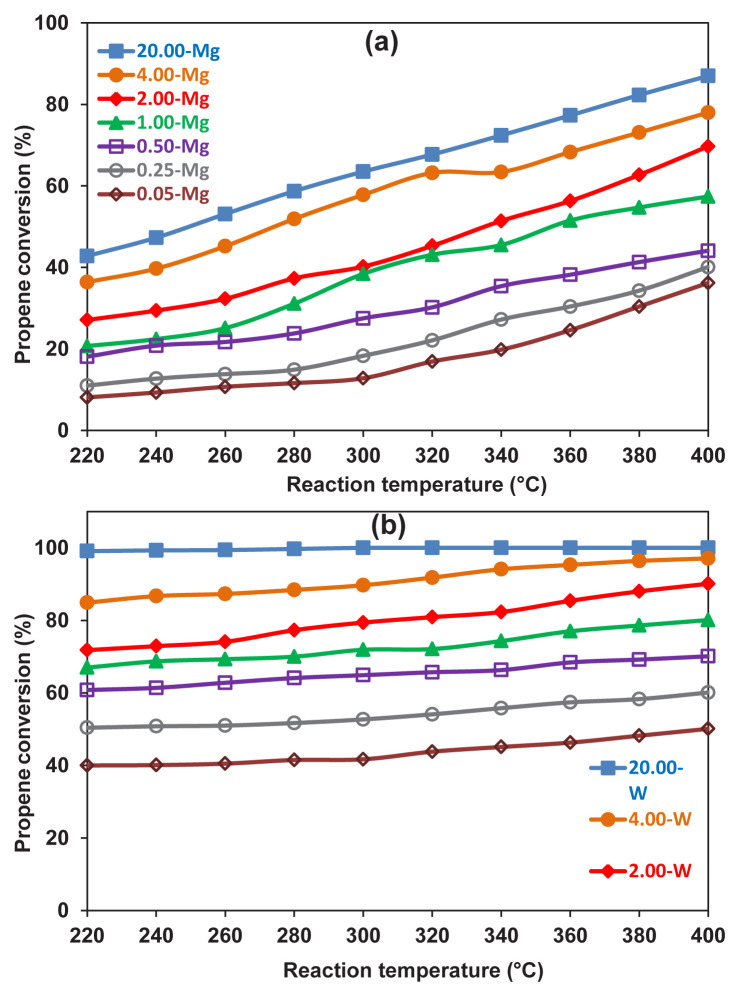
Effect of reaction temperature on the propene conversion of dehydration of propan-2-ol reaction over a) AMMO and b) AMWO catalysts.

Moreover, the results showed an increase in the conversion of propan-2-ol to propene with increasing temperature for AMMO (Figure 11a) while temperature does not have a significant effect when AMWO catalysts are employed (Figure 11b). As previously mentioned, WO_3_ only reacts with Al_2_O_3_; thus, increasing the molar ratio of MoO_3_:Al_2_O_3_ increased the amount of unreacted WO_3_, which seems to enhance the catalytic activity solely. This result can be tracked on the sample 20-W.

## 4. Conclusion

A series of Al_2_O_3_-MoO_3_-MgO and Al_2_O_3_-MoO_3_-WO_3_ catalysts with varying MoO_3_:Al_2_O_3_ molar ratios were synthesized. Materials were characterized with XRD, FT-IR, and SEM-EDS. For both catalyst sets prepared from either WO_3_ or MgO, dominating MoO_3_ phases were identified. The formation of the agglomerates (50–200 μm) on the surface of the catalysts was detected through SEM images. The EDS analyses confirmed the targeted amounts of the elements for all catalysts. Meanwhile, strong and very strong acidic sites of the Brønsted and Lewis types were observed on the surfaces of the catalysts. Moreover, the acidic site strength was found to increase as a function of increasing MoO_3_:Al_2_O_3_ molar ratio for both systems. The relation between the acidic sites of the catalyst and the dehydration activity for the conversion of propan-2-ol into propene was verified. High conversion and selectivity towards propene was achieved. The catalytic activity of the catalysts prepared from MgO increases with temperature. However, for the catalysts prepared from WO_3_, the effect of MoO_3_:Al_2_O_3_ molar ratio was found to be more critical rather than temperature change.

## Figures and Tables

**Table 1:** The compositions and symbols of the catalysts.

AMMO catalyst			AMWO catalysts		
Sample symbol	MoO_3_:Al_2_O_3_ molar ratio	Sample composition (mol)	Sample symbol	MoO_3_:Al_2_O_3_ molar ratio	Sample composition (mol)
0.05-Mg-1	0.05	(0.25 MgO:0.05 MoO_3_:1.00 Al_2_O_3_)	0.05-W-1	0.05	(0.25 WO_3_:0.05 MoO_3_:1.00 Al_2_O_3_)
0.25-Mg-2	0.25	(0.25 MgO:0.25 MoO_3_:1.00 Al_2_O_3_)	0.25-W-2	0.25	(0.25 WO_3_:0.25 MoO_3_:1.00 Al_2_O_3_)
0.50-Mg-3	0.50	(0.25 MgO:0.50 MoO_3_:1.00 Al_2_O_3_)	0.50-W-3	0.50	(0.25 WO_3_:0.50 MoO_3_:1.00 Al_2_O_3_)
1.00-Mg-4	1.00	(0.25 MgO:1.00 MoO_3_:1.00 Al_2_O_3_)	1.00-W-4	1.00	(0.25 WO_3_:1.00 MoO_3_:1.00 Al_2_O_3_)
2.00-Mg-5	2.00	(0.25 MgO:1.00 MoO_3_:0.50 Al_2_O_3_)	2.00-W-5	2.00	(0.25 WO_3_:1.00 MoO_3_:0.50 Al_2_O_3_)
4.00-Mg-6	4.00	(0.25 MgO:1.00 MoO_3_:0.25 Al_2_O_3_)	4.00-W-6	4.00	(0.25 WO_3_:1.00 MoO_3_:0.25 Al_2_O_3_)
20.00-Mg-7	20.00	(0.25 MgO:1.00 MoO_3_:0.05 Al_2_O_3_)	20.00-W-7	20.00	(0.25 WO_3_:1.00 MoO_3_:0.05 Al_2_O_3_)

**Table 2:** The weight percentage (wt.%) of the elements in the synthesized samples.

Element (wt.%) Sample	O	Al	Mo	Mg	W	Total
***0.50-Mg theo***.	*41.30*	*29.35*	*26.09*	*3.26*		*100*
**0.50-Mg-zone1**	41.19	28.97	26.59	3.25		100
**0.50-Mg-zone2**	40.68	29.12	27.01	3.19		100
***1.00-Mg theo***.	*39.06*	*21.09*	*37.51*	*2.34*		*100*
**1.00-Mg-zone1**	37.40	21.41	39.00	2.19		100
**1.00-Mg-zone2**	38.63	19.10	40.05	2.22		100
***2.00-Mg theo***.	*34.52*	*13.71*	*48.73*	*3.04*		*100*
**2.00-Mg-zone1**	35.01	14.17	48.06	2.76		100
**2.00-Mg-zone2**	37.18	12.47	47.91	2.44		100
***0.50-W theo***.	*36.21*	*23.28*	*20.69*		*19.82*	*100*
**0.50-W-zone1**	38.21	21.28	19.69		20.82	100
**0.50-W-zone2**	35.14	24.17	21.64		19.05	100
***1.00-W theo***.	*35.53*	*17.76*	*31.58*		*15.13*	*100*
**1.00-W-zone1**	34.17	16.14	33.17		16.52	100
**1.00-W-zone2**	35.14	14.14	34.50		16.22	100
***2.00-W theo***.	*33.21*	*10.67*	*37.94*		*18.18*	*100*
**2.00-W-zone1**	32.19	11.65	37.94		18.22	100
**2.00-W-zone2**	33.38	10.84	35.14		20.64	100
**2.00-W-zone3**	34.17	10.19	36.45		19.19	100
